# Tension type headache is dead; long live chronic migraine!

**DOI:** 10.1186/1129-2377-14-S1-P48

**Published:** 2013-02-21

**Authors:** GE Elrington

**Affiliations:** 1 National Migraine Centre, UK

## 

Chronic Migraine (CM) was first defined in 2004. The definition was revised in 2006[1]. The definition allows occurence of both migraine and tension-type headache (TTH). This study analyses the Introduction of the new diagnosis of CM in one neurologist's practice. A patient database for all patients seen in consultation has been maintained since 1994. Total patient numbers are currently over 22,000. Data are collected and recorded at the time of first consultation and updated when the patient is reviewed. All relevant diagnoses are recorded; patients often have more than one diagnosis. Before the definition of CM, patients with other primary headache, and medication overuse headache (MOH), were diagnosed with both the primary headache, and MOH, when appropriate. This practice continued when coding for CM, despite the IHS definition excluding CM in MOH patients. A steady rise in CM since 2004 has peaked at about 150 cases a year. This is mirrored by a fall in diagnosis of TTH to single figures annually. It is likely that patients with chronic headache,formerly diagnosed as both TTH and migraine, can now receive a single diagnosis of CM. CM has proved common in this practice and has largely superceded the diagnosis of TTH.

## Competing interests

The author has worked with most of not all of the pharma industry, including Allergan for whom he was a triallist in the PREEMPT study, and member of advisory boards.
